# Security issues of the gold industry chain based on smart blockchain in the context of the Internet of Things

**DOI:** 10.1038/s41598-024-52274-2

**Published:** 2024-02-01

**Authors:** Jifei Zheng, Guisen Wang, Yuhan Zhang, Lei Chen, Xiao Li, Shouting Zhang

**Affiliations:** 1 School of Earth Sciences and Resources, China University of Geosciences, Beijing, 100083 China; 2 Shandong Gold Group CO, LTD, Jinan, 250100 China; 3 Shandong Gold Group International Mining Development CO, LTD, Jinan, 250000 China; 4 Information Center, Ministry of Natural Resources PRC, Beijing, 10036 China; 5 Shandong Geological Survey Institute, Jinan, 250013 China

**Keywords:** Chemistry, Energy science and technology, Engineering, Materials science, Mathematics and computing, Nanoscience and technology, Optics and photonics, Physics

## Abstract

The purpose is to solve the safety production and management problems of the gold Industrial Chain and give early warning of the safety situation of the gold Industrial Chain. According to the theory of industrial chain security governance and the basic situation of the gold Industrial Chain, this work establishes a gold Industrial Chain model based on smart blockchain and system dynamics (SD), and discusses the application of the gold Industrial Chain in the Internet of Things (IoT) environment. The overall goal of the application of IoT technology to the safety management of intelligent gold mines is to take the gold mine production demand as the driving force. The digitalization of production, electromechanical, safety, dispatching, and other information realizes intelligent digital perception, assists decision-making, guides the safety management of gold mining operations, continuously improves the operation efficiency of the gold mining industry, and drives the development of the industry. Finally, it takes the resource reserve of China’s gold industry from 2011 to 2021 as the research data introduces the weighting method to assess the security situation of China’s gold Industrial Chain from 2011 to 2021. The safety performance of China's environmental industry chain is evaluated through the detailed introduction of the basic information of the gold Industrial Chain. The result shows that the security situation of China’s gold Industrial Chain from 2011 to 2021 shows an overall growth trend, 88.42% higher than in 2014. The security situation of China’s gold Industrial Chain from 2011 to 2021 positively impacted the opening of the domestic gold market and entering the international gold market, improving the security level of China’s gold Industrial Chain. In this work, a gold Industrial Chain security model based on smart blockchain and SD is established to solve the safety problem of the gold Industrial Chain, which can improve the safety level of the gold industry and promote its sustainable development.

## Introduction

With the development of the global economy and the constant changes in the financial market, gold, as an important hedge asset and store of value, plays an important role in the world. The Gold Industrial Chain covers the mining, processing, storage, circulation, and other links of gold, and there are various safety problems in this complex industrial chain. The safety of the gold Industrial Chain has a vital impact on the gold industry and related economic activities. In the process of gold mining and gold processing, there are theft, forgery, leakage, transportation safety, and other problems, and these security risks will seriously affect the stability and security of the gold Industrial Chain^1–3^. It is understood that in 2021, China’s gold output reached 328.98 tons, down 9.95% year on year (y/y). Among them, the domestic gold output of large gold enterprises (groups) accounted for 47.14% of the national total. In the first half of 2022, the gold production enterprises in Shandong Province, China’s largest gold-producing province, achieved remarkable results in resuming production. The output of gold in mines rose sharply, driving the national gold output to increase significantly. In the first half of the year, China’s gold output was 174.69 tons, up 14.36% y/y. Among them, the domestic mines and mines of large gold enterprises (groups) accounted for 52.85% of the national gold output. The industry concentration also recovered further^4^.

With the continuous progress of science and technology, emerging technologies such as smart blockchain and the Internet of Things (IoT) are gradually applied to the security management of the industrial chain. The decentralization and tamper-proof properties of smart blockchain technology guarantee the data security of the gold Industrial Chain. The application of IoT technology can realize the digital and intelligent management of all links in the gold Industrial Chain and improve the efficiency and safety of the gold industry. Aiming at the security issues of the gold Industrial Chain, combined with cutting-edge technologies such as smart blockchain and system dynamics (SD), gold Industrial Chain security in an IoT environment is studied. In-depth research and exploration are of great significance to improve the safety level of the gold industry and promote the sustainable development of the gold Industrial Chain^5–7^. During the exploration stage, IoT technology monitors underground rocks and strata in real-time through geological sensors and seismic monitoring equipment, helping exploration personnel accurately locate gold deposits. During the mining stage, intelligent mining is equipped with sensors to monitor the status and operation of the equipment to predict maintenance needs in advance, thereby improving equipment efficiency and extending service life. During the smelting process, IoT sensors monitor temperature, pressure, and chemical composition to ensure the quality and efficiency of gold extraction. In terms of logistics and supply chain management (SCM), radio frequency identification (RFID) tags and sensors can be used for tracking and secure delivery of gold goods, while vehicle tracking helps with route optimization and security management. Finally, during the sales and appraisal stages, blockchain technology ensures the traceability of transaction records and gold authenticity, while intelligent warehouses monitor storage conditions to guarantee the quality and safety of gold.

This work aims to solve the safety problems existing in the safe production and management of the gold Industrial Chain, and realize its early warning of the safety situation. Firstly, the theory of the Industrial Chain’s Security Governance and the basic situation of the gold Industrial Chain are discussed, to provide a theoretical basis. Secondly, on account of SD theory and smart blockchain technology, a gold Industrial Chain security model based on smart blockchain and SD is established, and the application of gold Industrial Chain security in an IoT environment is expounded. Finally, taking the resource reserve of China’s gold industry from 2011 to 2021 as the research data, the weighting method is adopted to evaluate the security situation of China’s gold Industrial Chain from 2011 to 2021. Moreover, the gold Industrial Chain security model based on the SD model under the guidance of IoT is verified. The innovations of this work are as follows: Taking the security situation of China's gold Industrial Chain as the research object, the dynamic process of the gold Industrial Chain is simulated and analyzed by combining smart blockchain technology and the SD theoretical model. Besides, IoT is used to collect, store and transmit data of the gold Industrial Chain to realize early warning of the security situation of the gold Industrial Chain.

The main content of this work is divided into five parts. “Introduction” section principally describes the current safety issues of the gold Industrial Chain, which provides the research background, idea, method process, and organizational framework for this work. “Literature review” section is the literature review, which mainly analyzes and discusses the application research of SD by scholars, IoT technology in industrial chain security, mineral resources supply prediction, and smart blockchain technology in data analysis security, to illustrate the necessity of this work. “Materials and methods” section is the research materials and methods, majorly explaining the Industrial Chain security governance theory, the basic situation of the gold Industrial Chain, smart blockchain technology, SD model, and IoT application in gold Industrial Chain security, thus providing material and basic theory for the analysis of gold Industrial Chain security model of smart blockchain and SD. “Results and discussion” section is the result and discussion, which chiefly evaluates the security situation of China’s gold Industrial Chain from 2011 to 2021. Under the guidance of IoT, the gold Industrial Chain security model on the basis of smart blockchain and SD is verified. “Conclusion” section is the conclusion, which primarily summarizes the research of this work and explains the research results, significance, deficiencies, and future development direction.

## Literature review

Since 1970, foreign countries have applied advanced technology to the intelligent mining industry, developing underground mining towards remote and autonomous control. China’s trackless mining equipment focuses on the R&D and improvement of mechanization, large-scale, and serialization. The research on equipment automation and intelligence has just started. Compared with developed countries, the technical level is relatively low. On the application of the System Dynamics Model (SDM), Nazari-Sharabian et al. described the application fields of SDM in three aspects: inference, management, and control optimization^8^. Cosenz et al. focused on the application of SDM in innovation systems and summarized its application in natural resource simulation and prediction research in land Resource Carrying Capacity (RCC), energy, mineral resources, and water resources^9^. Bao et al. used the SDM to simulate the mining city resources and environmental carrying capacity^10^. Zhang et al. established an SDM to restore and reconstruct the ecological environment in agro-pastoral ecotone along the Great Wall. A simulation study with relevant regional policies was conducted to obtain the best ecological environment governance model^11^. Furthermore, Shao et al. studied the relationship between water resources, environmental change, and socioeconomic systems based on the SD theory. The results showed that different levels of environmental change could impact the social economy of the Weihe River region in China ^12^. Audzijonyte et al. applied the SDM to study the marine ecosystem and explained each factor’s spatial and temporal distribution characteristics^13^. Machine learning and algorithms were increasingly used in SCM to handle large-scale and complex data. These models can automatically learn and predict demand, inventory levels, and delivery times in the supply chain. Aryal et al.^14^ adopted Leximancer's latent semantic analysis method, which could achieve faster, more reliable, and consistent content analysis, thereby achieving predictive SCM analysis. Dubey et al.^15^ explored SCM problems using a variance-based structural equation model, transforming the theoretical model into a statistical model that included the relationship between observed variables (actual measured values) and potential variables (concepts or constructs that cannot be directly observed). A variance–covariance matrix can represent these relationships.

In the safety research of the IoT in the Industrial Chain, Chedea et al. applied the SDM and the IoT to establish an early warning model for organic vegetable pests and diseases and carbon sink estimation. IoT could automatically monitor the pests and diseases in organic vegetables and the carbon sink process thanks to the low-cost and high-frequency data transmission characteristics of Wireless Sensor Networks (WSNs)^16^. Zhao et al. established underground micro-energy harvesting using WSNs and IoT technology. By testing the light intensity of several mine roadways, the feasibility of underground weak light energy- > electricity conversion and WSN-based power supply schemes have been verified^17^. Iftekhar et al. established a supervision system for the whole industrial chain of native chicken based on IoT and RFID technology^18^. They found that IoT and RFID technology can transmit transparent, visual and verifiable industrial chain information to ensure the production safety of agricultural products. Sharma et al. conducted a detailed analysis of the strengths and weaknesses of the industrial chain in the IoT background with the Strengths, Weaknesses, Opportunities, Threats (SWOT) analysis^19^. They found that IoT technology was conducive to establishing industrial chain platforms by the government, education, and other institutions. Latif et al. combined IoT and blockchain technology to build an electronic voting system^20^, which solved the problems of low efficiency and complex operation of the traditional electronic voting system, improved the robustness of the voting system, and realized effective monitoring of the electronic voting system.

Concerning the research of smart blockchain technology in data analysis security, Ding et al. conducted a detailed analysis of the current financial industry data crisis from the perspective of intelligence analysis. They used human–computer intelligence and blockchain technology to build a financial information security analysis model. The proposed model improved the maintenance of financial security and early warning of a financial crisis^21^. Lu et al. established a crop whole-industry chain information traceability platform based on the credit-supervisor byzantine fault tolerance (CSBFT) blockchain and found that CSBFT has a low delay and higher security on the crop information traceability platform^22^. Ada et al. studied the application of blockchain technology in the automobile industry chain and found that the application of blockchain technology in the automobile industry chain can ensure the information security of the automobile industry chain management and promote the development of the automobile industry chain^23^. Boakye et al. reconstructed the technical structure of private equity crowdfunding platform on the basis of blockchain technology^24^ and found that blockchain technology was integrated into the form of an alliance chain, which improved the efficiency of the equity trading platform. Trivedi et al. established the industrial chain ecosystem of consumer finance security from the perspective of consumer financial security combined with blockchain technology^25^.

Based on the literature review, previous studies have explored the security and management of the gold Industrial Chain by applying smart blockchain, SD, and IoT technology. However, there are still some defects in these studies, and the technical level of the gold Industrial Chain in intellectualization, automation, and informatization is relatively low. Therefore, research can focus on technological innovation and upgrading to achieve intelligent gold Industrial Chain security management. Existing static indicators have limitations in reflecting the dynamic supply of mineral resources such as fossil fuels or metal minerals. Consequently, the research can improve the supply forecasting ability of the gold Industrial Chain by introducing dynamic models and forecasting methods. To sum up, this work combines smart blockchain and IoT technology to ensure data credibility and security of each link in the supply chain through smart blockchain technology, providing traceability and transparency, thus reducing supply chain risks and guaranteeing the safety of the gold Industrial Chain. IoT technology is used to strengthen the transparent, visual, and verifiable information transmission in the gold Industrial Chain, ensure the security of information transmission, and promote the development of the entire industry in the direction of intelligence and security. Moreover, mining processes' automation, remoteness, and intelligence in the gold Industrial Chain are realized to improve the production efficiency and safety.

## Materials and methods

### Theory of Industrial Chain security governance

**Figure 1:**
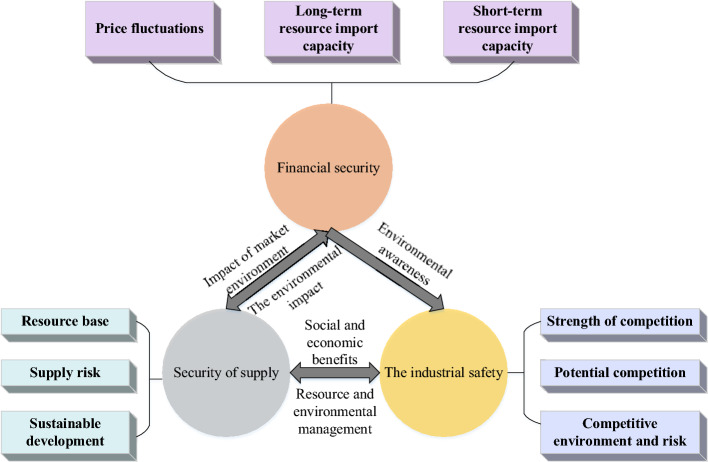
System model of national O&G resources security and sustainable development.

Regarding the governance evaluation theory of the gold Industrial Chain’s security situation, Dale et al. explored the reasons behind the changes in the national Oil and Gas (O&G) security situation. They argued that the evolution of O&G resources lies in the changes in the financial industry and market competitiveness brought about by the supply & demand imbalance of O&G resources. A model of a sustainable development system for national O&G resource security was proposed^26^. Figure 1 draws a model for the national O&G resources security sustainable development system.

**Figure 2:**
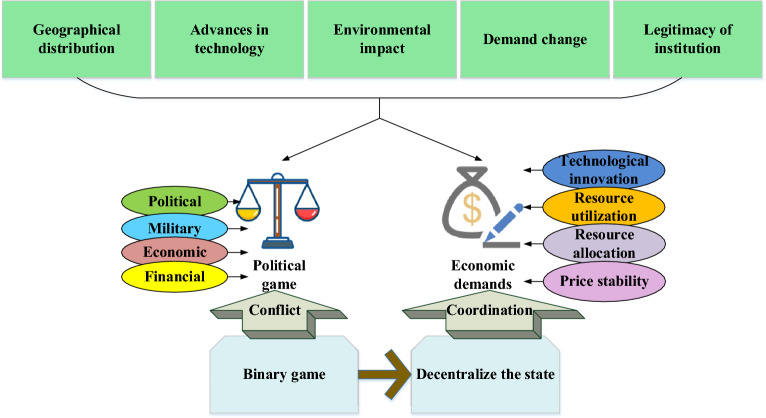
Evolution of resource-oriented Security Governance Theory.

In Fig. 1, Due to the imbalance of supply and demand caused by the change of external dependence, the comprehensive effect of resources, political, economic, military, and geopolitical forces is highlighted. With the further advancement of sustainable development of the mining industry chain, the negative environmental externalities of O&G resource development and the socioeconomic development imbalance have attracted extensive attention. Consequently, technology development, energy efficiency improvement, resource structure, environmental impact, and sustainable development of the resource Industrial Chain’s security assessment have been included in the supply security assessment system. The further integration of globalization and the elements of global value chain governance profoundly impact the distribution of resources. Resource security management not only restricts resource allocation but also is closely related to technological development, environmental changes, supply and demand changes, and the legitimacy of the system. Thereupon, resource security gradually presents a binary game from resource producers and consumers to the centralized management of the country, showing the diversified development of participants, extensive management objects, and multi-level management objectives^27^. Figure 2 shows the evolution of resource-oriented Security Governance theory.

**Figure 3:**
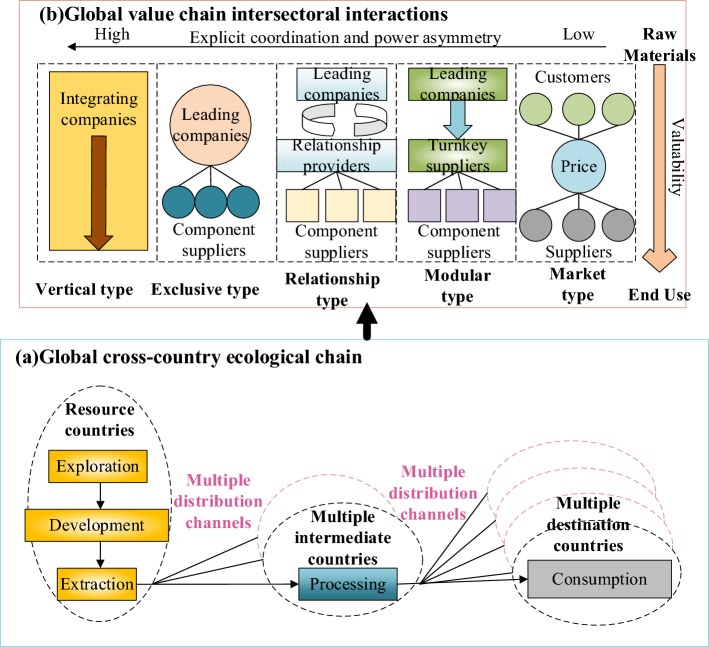
Governance model of the global Industrial Chain.

Figure 2 is the evolution of resource-oriented Security Governance theory, where the governance core has shifted from a political game to economic supply and demand coordination. The governance subject has changed from national sovereignty to multi-subject participation. The governance object has expanded from mineral resources to technical optimization, and new technology, materials, and industries have been fully considered. The governance goal has evolved from ensuring production safety channels to adopting green development under sustainable development. As for the research on the value governance of Industrial Chains, Pananond et al. chose the inter-enterprise industrial organization and divided the governance of global Industrial Chains into market-oriented, modular, relational, exclusive, and vertical models based on transaction complexity, information coding, and supplier capabilities^28^. Figure 3 plots the governance model of the global Industrial Chain.

Figure 3 displays that the global industrial chain governance model relies on transaction cost economics for global value chain governance. Several intermediate countries enter global value chains controlled by leading firms. By undertaking industrial transfer, the three-stage development of the global transnational production chain has been completed, the domestic technological change has been realized. Additionally, the function upgrading of the global value chain has been finished, and the economic development of developed countries has been attempted to achieve convergence. Led by market forces, leading companies lock emerging economies such as China into the lower end of the value chain through rules and regulations. It is difficult for enterprises in multiple intermediate countries to enter the higher end of the global value chain controlled by developed countries. The governance model of the global industrial chain is based on the interaction between the global value chain departments (enterprises and components\material suppliers), coordination, and asymmetry of rights. The global transnational production chain is to obtain resources from exploration, development, and extraction of resources, thereby entering multiple distribution channels, multiple intermediate countries to obtain resource processing, and multiple purposes to consume resources.

### Basic information on the gold Industrial Chain

**Figure 4:**
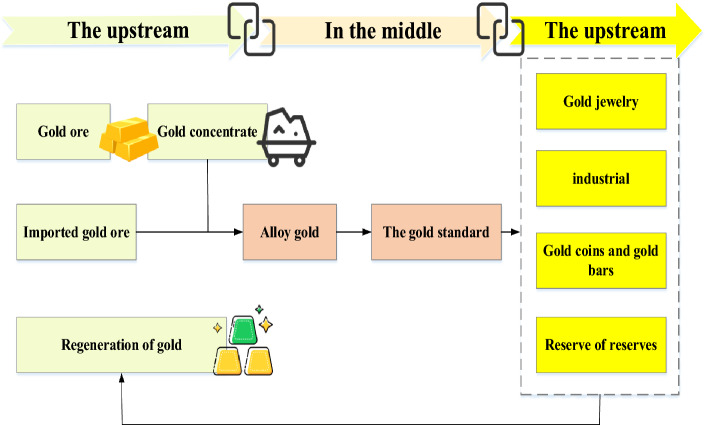
Gold Industrial Chain.

China’s gold market has formed a multi-level, multi-form, and multi-functional demand system for gold processing and manufacturing, wholesale and retail, lease financing, asset allocation, and investment and trade^29^. Figure 4 is the flow of a gold Industrial Chain.

From the gold Industrial Chain perspective in Fig. 4, gold’s upstream, middle reaches, and downstream involve the mining industry, the smelting industry, and the gold jewelry (and gold industry and reserves), respectively. While gold sales and industrial gold are in consumer demand, gold coins, bars, and reserves are in financial demand. Besides, gold smelting & recycling is also an essential part of the industry.

**Figure 5:**
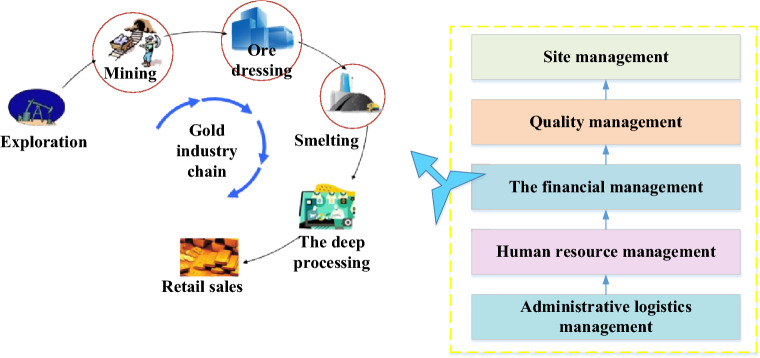
Value chain distribution of the gold industry.

China’s gold industry involves multiple participants, mainly comprehensive enterprises. Most gold enterprises cover multiple links and subdivided products in gold’s upper and middle reaches. The gold Industrial Chain’s profitability distribution is extremely uneven from link to link, as explained in Fig. 5.

Apparently, exploitation, beneficiation, and smelting are the core value chain links of the gold industry and the main factors affecting the gold production cost^30^. Thus, optimizing these three links can directly maximize the cost of gold production. Overall, the upstream production and material selection enterprises gain the utmost profitability in the chain, and some differential enterprises in the downstream processing and sales links also have strong profitability. The profitability of the smelting in the middle reaches the most meager, and it is in the break-even balance or marginal-profit state. The upstream mining and dressing enterprises are restricted by access qualification, mineral resources, and capital investment, with the highest concentration, and the gross profit rate can generally reach 50–60%.

### The mechanism of smart blockchain technology in the gold Industrial Chain security system

Smart blockchain technology provides a reliable, secure, transparent, and efficient operating environment for the gold Industrial Chain's security system through mechanisms such as data security, traceability, smart contracts, de-trust, and enhanced security governance. Thereby, smart blockchain technology plays an important role in the gold Industrial Chain security system, and its main mechanism includes the following aspects:

i. Smart blockchain ensures data security and integrity in the gold Industrial Chain through the use of decentralized distributed ledgers and cryptography algorithms. Each block contains the previous block’s hash value, making tampering with the data very difficult. Any attempt to tamper with the data will be detected by other nodes, thus protecting the security of the gold Industrial Chain.
ii. Smart blockchain technology enables data traceability and transparency across the gold Industrial Chain. Every transaction and operation is recorded on the blockchain, tracing back to when it happened and who was involved. This transparency enables any unusual activity or violation to be tracked and detected, thereby enhancing the security of the gold Industrial Chain.
iii. Smart blockchain technology enables automated security mechanisms through smart contracts. Smart contracts are blockchain-based programmable code that automatically performs certain actions according to pre-set rules and conditions. In the gold Industrial Chain, smart contracts can be used to manage contracts, settle payments and monitor the supply chain, reducing the risk of intermediate links and the possibility of human intervention.
iv. Smart blockchain technology implements a mechanism of de-trust, enabling all participants in the gold Industrial Chain to conduct safe and reliable transactions and cooperation without completely trusting each other. Blockchain, as a shared distributed ledger, records the transactions and behaviors of all parties involved and ensures the credibility and security of transactions through algorithms and protocols.
v. Smart blockchain technology offers strong support for security governance and compliance of the gold Industrial Chain. Through smart contracts and data records on the blockchain, regulators and relevant parties can conduct supervision and audit more effectively. It ensures that operations in the gold Industrial Chain comply with regulatory requirements, and timely discover and deal with security risks.

The running code of smart blockchain technology in the gold Industrial Chain security system is exhibited in Table 1.

**Table 1:** The running code of smart blockchain technology in the gold Industrial Chain security system.

Start		Running code
Step 1	Initialize blockchain	initializeBlockchain()
Step 2	Create a gold Industrial Chain security system	createGoldSecuritySystem()
Step 3	Listening for new transaction requests	listenForNewTransactions()
Step 4	Verify the effectiveness and compliance of transactions	validateTransaction()
Step 5	Check if the transaction meets security rules	if (transactionMeetsSecurityRules())
Step 6	Add transaction to pending block	addToPendingTransactions()
Step 7	Mining process	if (shouldMineNewBlock())
Step 8	Start mining and create new blocks	mineNewBlock()
Step 9	Update blockchain status	updateBlockchainState()
Step 10	Check for malicious behavior or security vulnerabilities	if (hasMaliciousBehavior())
Step 11	Implement corresponding security measures to prevent malicious nodes from participating, etc	takeSecurityMeasures()
End		

This work uses smart blockchain technology’s intelligent and trusted computing process to simulate and analyze the dynamic process of the gold Industrial Chain. And IoT is employed to collect, store, and transmit data of the gold Industrial Chain to realize early warning of its security situation.

### Construction of gold Industrial Chain security model based on smart blockchain and SD

SD is a system simulation method proposed by Professor Forrester in 1956, used to analyze enterprise problems, such as production and inventory management^31^. Table 2 shows the basic concepts of SD.

**Table 2:** Basic concepts of SD.

Basic concepts	Explanations
System	Elements that are different from each other and interact with each other are organically connected to achieve specific functions of the same purpose
Feedback	Relationship between system output and external environment input
Feedback system	The system containing feedback links and their functions
Feedback loop	A closed path consisting of information and operations
Causal loop diagram	Arrow containing multiple variables and causality
The polarity of the causal chain	Positive (+) means the changing trend of the two variables is the same Negative (−) means the changing trend of the two variables is opposite
The polarity of the feedback loop	The positive feedback loop is the deviation enhancement of variables in the loop The negative feedback loop is the variable in the loop that tends to be stable
Method of determining loop polarity	If the feedback loop contains an even number of negative causal chains, the polarity is positive (+) If the feedback loop contains an odd number of negative causal chains, the polarity is negative (−)
System flow diagram	The graphic model of the interconnection form of each level variable and each rate variable in the feedback loop contains the interconnection relationship between the loops in the feedback system

**Figure 6:**
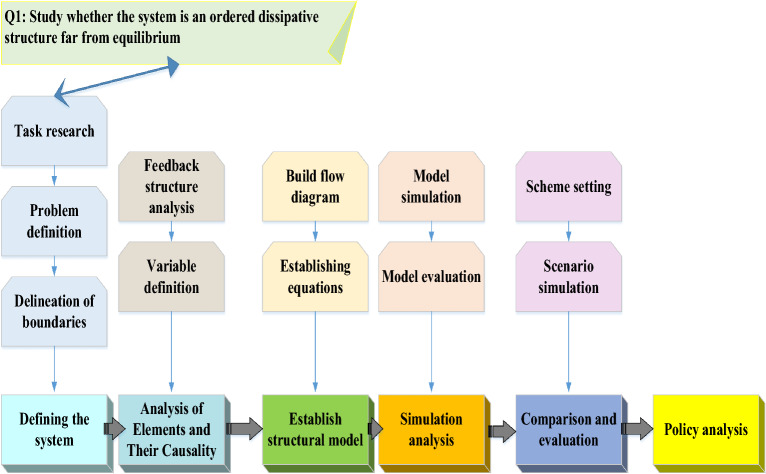
Modeling process of SD.

The problem-solving process in SD is essentially a process of seeking optimization for better system function. SD emphasizes the structure of the system and analyzes the function and behavior of the system from the perspective of system structure. The system structure determines the behavior of the system. Therefore, SD is to obtain the optimal behavior of the system by finding the optimal structure of the system. SD believes that the system is a causal feedback mechanism with multiple information. Hence, after analyzing the system and getting profound and rich information, the causality feedback diagram is established for the system and then converted into a system flow chart to establish SDM. Finally, the SDM is simulated by using simulation language and software, and the real system structure is simulated. The simulation of the system structure is completed through the above process. The next is to find a better system structure. Searching for a better system structure is called strategy analysis or optimization, involving parameter optimization, structure optimization, and boundary optimization. Parameter optimization changes the system structure by changing sensitive parameters to find the optimal system behavior. Structure optimization refers to changing the system structure by increasing or decreasing the horizontal and rate variables in the model to obtain better system behavior. Boundary optimization means that the system structure changes when the boundary and boundary conditions change to obtain better system behavior. SD is to simulate the system structure through computer simulation technology, find the optimal structure of the system, and thus obtain the optimal system behavior^32–34^. Figure 6 explains SDM’s modeling process.

Figure 6 before building the SDM, a clear understanding of the research object must be formed. The system studied must be an orderly dissipative structure far from equilibrium. Then, the system-dynamics analysis is performed on the research object to establish the causal relationship diagram and flow diagram of the design object elements and the problem’s scope. Simulation experiments and calculations are conducted using the system-dynamics equation^35^. Equations (1)–(3) are the dynamic equation of combined external force applied to the system:

1 $$ \sum F = m_{1} a_{1} + m_{2} a_{2} + m_{3} a_{3} + \cdots + m_{n} a_{n} $$

2 $$ \sum F_{x} = m_{1} a_{1x} + m_{2} a_{2x} + m_{3} a_{3x} + \cdots + m_{n} a_{nx} $$

3 $$ \sum F_{y} = m_{1} a_{1y} + m_{2} a_{2y} + m_{3} a_{3y} + \cdots + m_{n} a_{ny} . $$

In Eqs. (1)–(3), $m_{1} , \ldots , m_{n}$ is the mass of objects in the system. $a_{1} , \ldots , a_{n}$ represents the acceleration of objects in the system. $\sum F$ denotes the resultant external force on the system. $\sum F_{x}$ and $\sum F_{y}$ mean the resultant external force component of the system on the $x$-axis and $y$-axis, respectively. Equation (4) is the system-dynamics equation:

4 $$ L = L_{0} + \left( {R_{1} - R_{2} } \right) \cdot \Delta t. $$

In Eq. (4), $L$ represents the inventory of the system flow chart. $L_{0}$ is the initial inventory of the system flow chart. $R_{1}$ stands for system output rate. $R_{2}$ indicates the system delivery rate. $\Delta t$ refers to the system flow changes’ accumulation time.

**Figure 7:**
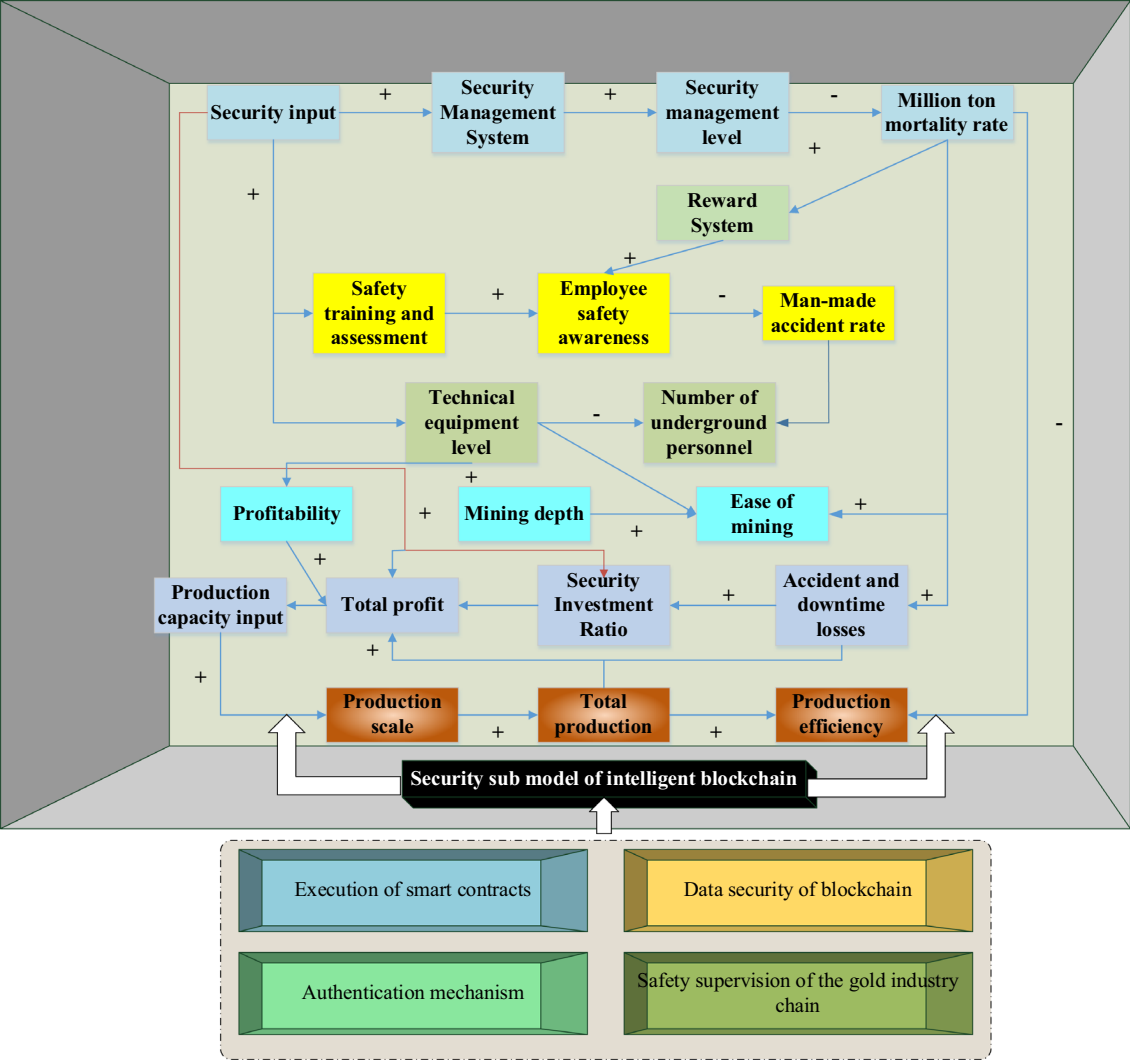
The gold Industrial Chain security model based on smart blockchain and SD.

The mechanism of SDM in the proposed system is to provide managers with a comprehensive system perspective and decision support to ensure the security and stability of the gold Industrial Chain. It is achieved through the application of dynamic modeling, risk assessment, early warning, strategy development and optimization, business decision support, and continuous improvement and optimization. SDM provides a systematic method to reveal the interdependence and complex dynamic behaviors among all links in the gold Industrial Chain, to help managers better understand the system's operating mechanism and evolution law. The mechanism of SDM in the gold Industrial Chain security system is as follows:

i. SDM can dynamically model the relationship and interaction of all links in the gold Industrial Chain, covering gold mining, processing, circulation, and other links. By considering the feedback and delay effects between each link, the model can more accurately describe the behavior and evolution process of the system.
ii. SDM can simulate and analyze the impact of different security risk factors on the system to assess the vulnerability and security risk level of the system. Based on the analysis of the model, the potential security problems can be warned in advance, and corresponding measures can be taken to reduce the risk.
iii. SDM can be used to evaluate and optimize the effectiveness of different security management policies and measures. Through the model analysis, the optimal security management strategy can be found, involving resource allocation, risk management, and monitoring measures, to improve the security of the gold Industrial Chain.
iv. SDM can provide decision-makers with insight into the system's overall operation and development trends. The model can help decision-makers understand the interaction between different factors, predict the development trend of the system, and make corresponding business decisions based on the information, thus optimizing the safety management of the gold Industrial Chain.
v. SDM is an iterative process that continuously improves and optimizes the accuracy and applicability of the model through the continuous collection and collation of actual data, and the calibration and validation of the model. This allows security management policies and measures to be constantly adjusted and improved according to the actual situation to cope with the ever-changing security risks. To sum up, a gold Industrial Chain security model based on smart blockchain and SD is implemented, as indicated in Fig. 7.

In Fig. 7, the factors in the gold Industrial Chain’s security SDM restrict and influence each other. There are multiple positive and negative feedback relationships. Specifically, positive feedback loop: technical facility level (+) the number of mining personnel (+) mine accident rate (+) mining mortality (+) production efficiency (+) output value (+) industrial profit (+) safety investment capital (+) technical facility level. Negative feedback loop: safety investment fund (−) safety training (−) employee safety awareness (−) mining accident rate (−) mine mortality (−) industrial mining accident loss and shutdown loss (−) safety investment. The feedback loop of the gold Industrial Chain’s security SDM starts from the relationship between the various factors. Then, it evaluates the gold Industrial Chain’s safety management level and safety training through the production scale of the gold industry, economic profits, safety and production investment, technological progress, equipment level, safety officers, and mining staff, simulating and analyzing the dynamic change and evolution of gold Industrial Chain. It is based on the security submodel of smart blockchain, including blockchain data security, authentication mechanism, smart contract enforcement, and regulation. Applying blockchain technology can enhance the security, authenticity, and traceability of information in the gold Industrial Chain.

### Application of IoT in the gold Industrial Chain’s security

**Figure 8:**
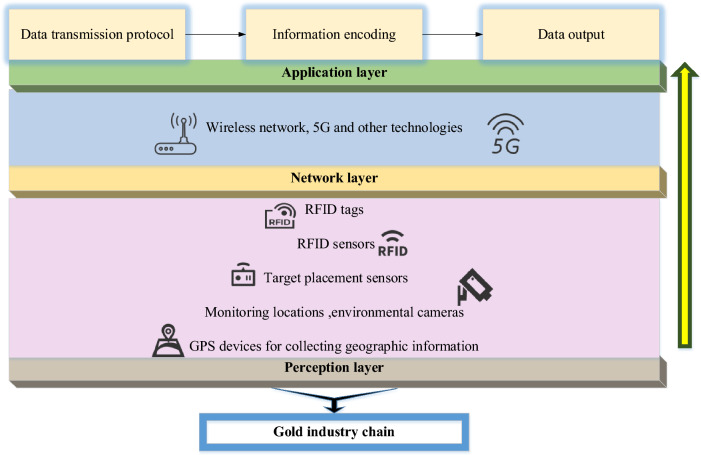
Application of IoT in gold Industrial Chain’s security management.

IoT provides real-time monitoring, traceability, and security management capabilities for gold Industrial Chain security systems by connecting and transmitting physical devices, sensors, and the Internet^36,37^. Figure 8 shows the application of the IoT in gold Industrial Chain security management.

Figure 8 signifies that the perception layer can be used to monitor and collect data related to gold production, transportation, and storage. By means of temperature, humidity, and vibration sensors, gold's environmental conditions and transportation state can be monitored in real-time to ensure the safety and quality of gold. The network layer can use various communication technologies and protocols to establish reliable and secure data transmission channels. The Wireless Sensor Network (WSN) of IoT or IoT protocol stack technology transfers the data collected by the perception layer to the subsequent application layer for processing and analysis. The application layer of IoT is the level of processing, analysis, and application of data at the perception and network layers. In the gold Industrial Chain security system, the application layer can monitor and analyze the real-time data the perception layer collects. Setting warning rules and thresholds allows potential security problems to be discovered and warned in time.

### Data source and experimental environment

To study the security of the proposed IoT-based gold Industrial Chain’s security SDM, this section takes the resource reserve of China’s gold industry from 2011 to 2021 as the research data. The data source is the National Bureau of Statistics (NBS). Data collection is a key part of this work. The data is sourced from the NBS, and the agency has certified its quality. The data involves historical data on gold resource reserves, covering 2011 to 2021. These data are considered the basis of this work to analyze the safety of the gold Industrial Chain.

Then, it refers to the literature of Tan et al. to assess the security situation of China’s gold Industrial Chain^38^. Therefore, China’s gold Industrial Chain security is calculated by Eq. (5):

5 $$ GSI = \frac{GSR + DES + DC}{3}. $$

In Eq. (5), $GSI$ (Gold Safety Industry) represents China’s gold Industrial Chain’s security index. $GSR$ (Gold Stability Resource) is the stability of global resource supply. $DES$ (Domestic Economic Security) denotes domestic economic security, and $DC$ (Dominant Coexistence) stands for domestic economic security.

The construction and simulation of SDM rely on the new version of the SD professional software Vensim. Vensim runs on the Windows operating system, and its installation process includes copying the installation files and Vensim system files to the hard drive and then clicking on the installation file according to the prompts to install.

Experimental environment: The hardware configuration used in this work is Intel Core i5-7200U CPU@2.50 GHz; Memory (Random Access Memory): 8.00 GB, Central Processing Unit (CPU): NVIDIA GeForce 94MX. The operating system is Windows 10 64-bit. This hardware environment provides sufficient computing power and resource support for the operation of the SDM to conduct SD simulations of the gold Industrial Chain security.

This work adopts the supply chain security framework as the foundation of the governance theoretical framework, which covers various aspects of the supply chain, including suppliers, logistics, information flow, and financial flow. This framework provides a theoretical basis for analyzing the security of the gold Industrial Chain and helps identify key risk factors. Smart blockchain and SD methods are chosen because they allow for establishing a multi-level, dynamic model that can capture complex interactions in the supply chain. This aligns with the theoretical framework of supply chain security governance, as it emphasizes the connections and interdependence between various nodes in the supply chain.

## Results and discussion

### Evaluation of china’s gold Industrial Chain’s security situation

**Figure 9:**
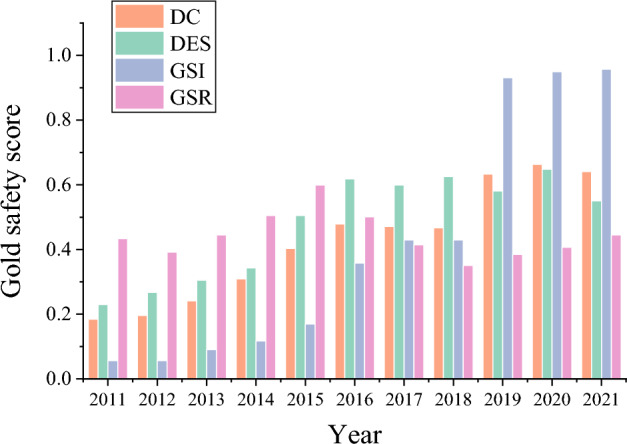
Score of China’s gold Industrial Chain’s security situation from 2011 to 2021.

The security score of China's gold Industrial Chain under different security situation indexes from 2011 to 2021 is portrayed in Fig. 9. Apparently, the security situation from 2011 to 2021 shows an overall growth trend. In 2021, the security index of China’s gold Industrial Chain reached 0.95, 88.42% higher than that of 2014. The security level of China’s gold Industrial Chain has increased to varying degrees in terms of $GSR$, $DES$, and $DC$. The $DES$ and $DC$ indexes have increased by 46.88% and 50.77%, respectively. The data show that the security situation of China’s gold Industrial Chain from 2011 to 2021 positively impacts the opening of the domestic and international gold markets, thus improving overall security.

**Figure 10:**
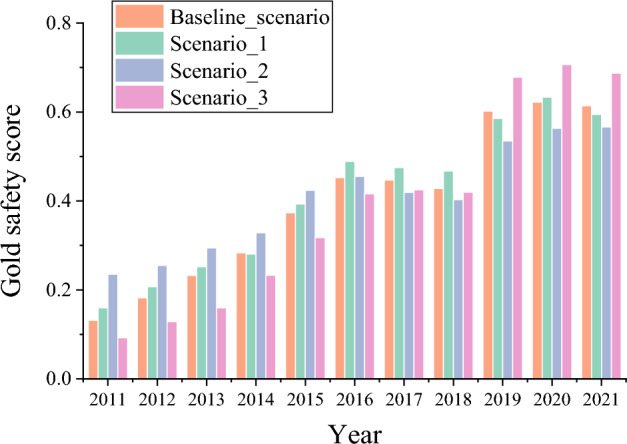
Safety index of China’s gold Industrial Chain under different scenarios.

Subsequently, to clarify the role of $GSR$, $DES$, and $DC$ in the security situation of China’s gold Industrial Chain, this section uses the Monte Carlo simulation method to assign the maximum weight to $GSR$, $DES$, and $DC$, respectively. In each scenario, 10,000 iterations are completed to obtain the median gold security score. Scenario 1: GSR has greater weight than DES and DC, emphasizing the role of global resource supply stability in the gold Industrial Chain’s security. Scenario 2: DES has greater weight than GSR and DC, emphasizing the role of domestic economic security in the gold Industrial Chain’s security. Scenario 3: DC has greater weight than GSR and DES, emphasizing the role of superior coexistence in the gold Industrial Chain’s security. Figure 10 shows the safety index of China’s gold Industrial Chain under different scenarios.

Clearly, the absolute increase of the safety index of Scenario 1 is lower than that of Scenario 3 and higher than that of Scenario 2, and the gap with the basic scheme is the smallest. The data imply that the safety index of China’s gold Industrial Chain increased by the largest margin from 2019 to 2021 when the coexistence of superior states is given the maximum weight. Thus, the coexistence of superior states plays an important role in the safety of the gold Industrial Chain. While the stability of the global resource supply and the security of the domestic economy are given the maximum weight, the security index of China’s gold Industrial Chain has increased to varying degrees since 2015. This shows that the stability of the global resource supply and the security of the domestic economy play an important role in the security of the gold Industrial Chain. The improvement of domestic economic security of China’s gold industry resources positively impacts the security level of China’s gold Industrial Chain in the short term. However, in the long run, domestic resource economic security is the defect of improving the security of China’s gold Industrial Chain.

### Analysis of the IoT-based gold Industrial Chain’s security SDM

**Figure 11:**
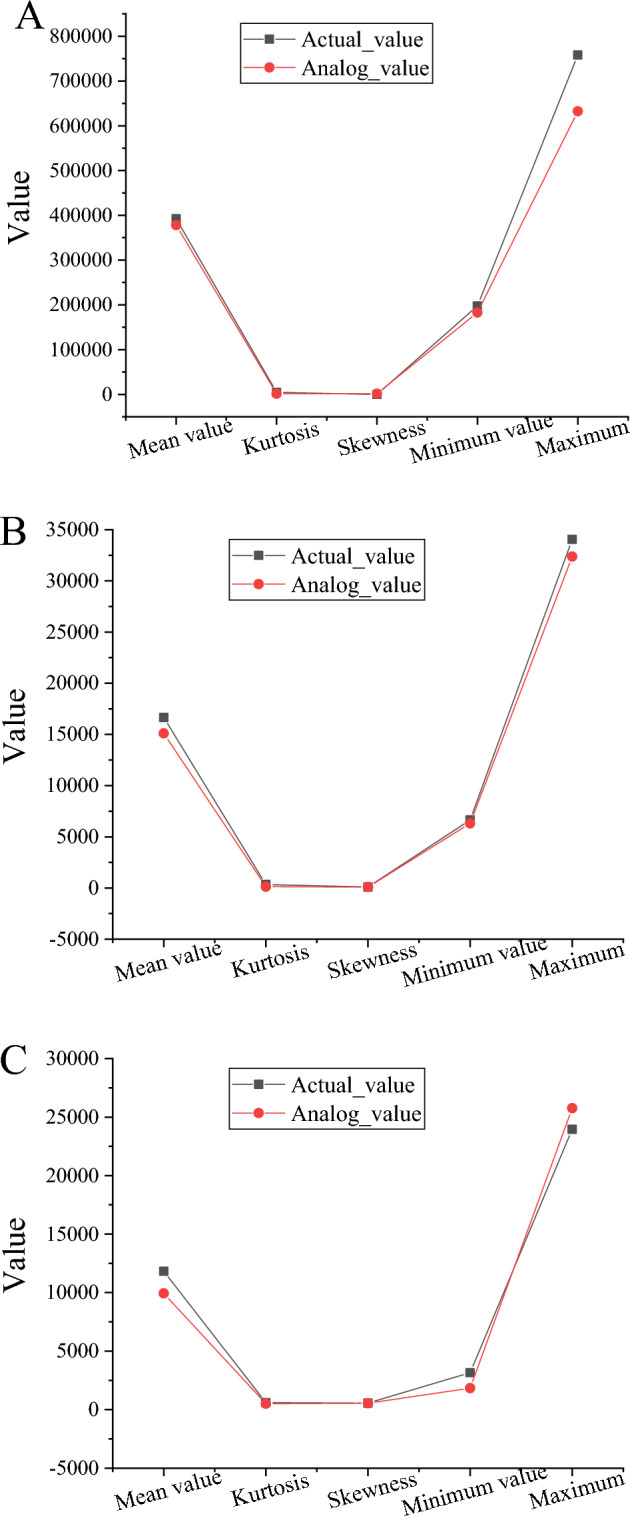
The verification results of the main variables of the model from 2011 to 2021 [(**A**) price indicators, (**B**) gold consumption, and (**C**) net imports of gold raw materials].

Figure 11 verifies the main variables of the model (gold price, net gold imports, demand, gold consumption, and production capacity) from 2011 to 2021. Figure 11a shows the change in the price index data of the model from 2011 to 2021, Fig. 11b shows the change in the gold consumption index from 2011 to 2021, and Fig. 11c shows the change in the net import data of gold raw materials from 2011 to 2021.

Figure 11 suggests that the gap between the actual and simulated values of each statistical indicator is gradually decreasing. The actual and simulated values of the proposed IoT-based gold Industrial Chain’s security SDM are close to each other regarding the gold price, gold consumption, and net import of gold raw materials. It shows that the simulation results are excellent and can genuinely reflect the security situation of the gold Industrial Chain. In the aspect of price indicators, the similarity between the proposed model’s simulated and actual values is 96.96%. Regarding gold consumption indicators, the similarity between the actual and simulated values of the model is 92.16%. Regarding the net import of gold raw materials, the similarity between the actual and simulated values of the model is 83.69%.

### Security, transaction efficiency, and system stability analysis of gold Industrial Chain security model of smart blockchain and SD

**Figure 12:**
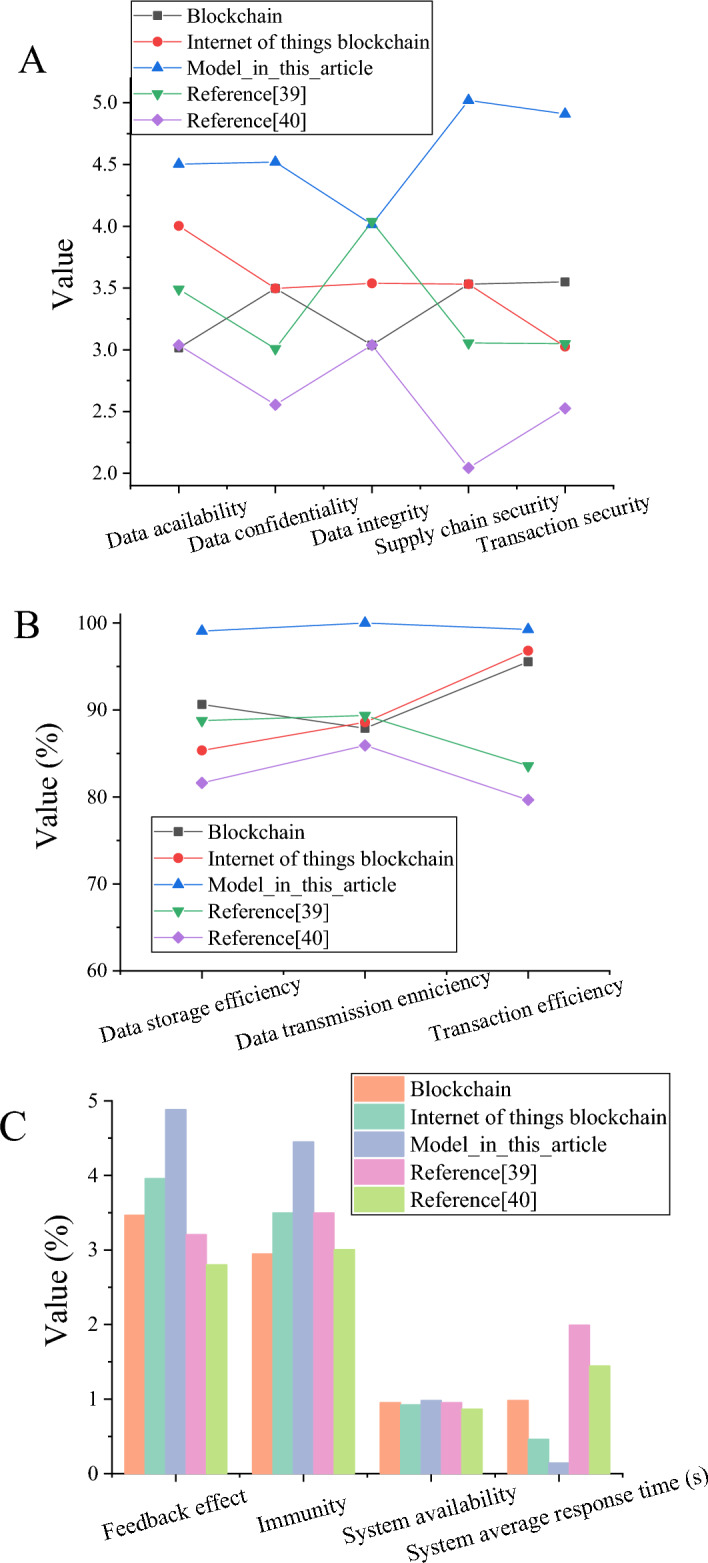
The evaluation results of the smart blockchain and SD’s gold industrial chain security model [(**A**) security; (**B**) transaction efficiency; (**C**) system stability].

To evaluate the security, transaction efficiency, and system stability of the gold Industrial Chain security model of smart blockchain and SD, this work applies blockchain in the gold Industrial Chain security system. IoT + blockchain respectively demonstrates gold Industrial Chain security, transaction efficiency, and system stability. In addition, Kalantari et al. applied meta-synthesis and fuzzy Delphi method for information security construction^39^, and Ghazal et al. used machine learning technology for information security construction of supply chain information collaboration. The use indicates the security degree, system anti-interference, and system feedback effect of the gold Industrial Chain security system^40^. The evaluation results of smart blockchain and SD’s gold Industrial Chain security model are suggested in Fig. 12.

Figure 12 shows that the proposed model has the highest score of 4.58 in data security, while other models have relatively low scores of 3.3, 3.5, and 2.6, respectively. This means that the proposed model based on smart blockchain and SD can effectively protect data confidentiality, ensure data integrity and availability, and ensure that unauthorized access does not leak data. The proposed model has a high evaluation score regarding transaction, data storage, and data transmission efficiency, thus providing efficient and reliable gold Industrial Chain operation and data processing capabilities. The proposed model has an anti-jamming capability of 4.5. In contrast, the other models score relatively low at 3, 3.5, 3.5, and 3, respectively. The proposed model can deal with all kinds of interference and attack to ensure the security and stability of the system. In addition, this model system's average response time, system availability, and feedback effect show high evaluation scores. Furthermore, it can provide stable, efficient, and reliable services to meet users’ needs. The proposed model has a high evaluation score in terms of transaction efficiency, data storage efficiency, and data transmission efficiency, To provide efficient and reliable gold Industrial Chain operation and data processing capabilities.

## Conclusion

This work surveys the resource reserve of China’s gold industry from 2011 to 2021 and uses the weighting method to assess the security situation of China’s gold Industrial Chain from 2011 to 2021. The gold Industrial Chain security model of smart blockchain and SD is also evaluated. The research results show that the security situation of China’s gold Industrial Chain from 2011 to 2021 positively impacted the opening of the domestic and the international gold market, improving the overall security level. Improving the domestic economic security of China’s gold industry resources positively impacts the security level of China’s gold Industrial Chain in the short term. Meantime, the management system of the smart blockchain is employed to design the security management mechanism of the gold Industrial Chain. Based on the SD theory, the safety performance of the gold Industrial Chain is evaluated. On the basis of the weight of the model data, the safety index of the industry chain is designed. The gold industrial chain security model based on IoT and SD has a similarity of 96.96% between the model’s actual and the simulated values of the gold price index. In the index of gold consumption and gold raw material net import, the similarity between the two values is 92.16% and 83.69%. The actual value is close to the simulated value. The simulation results are excellent, reflecting the true situation of the gold Industrial Chain. The finding provides a reference for developing China’s gold Industrial Chain and promoting its security. The gold Industrial Chain security model based on smart blockchain and SD shows good security and system stability features and has certain advantages in transaction efficiency. The IoT + blockchain model still needs to be further optimized and improved in some aspects. However, there are still some shortcomings. For example, the security situation of China’s gold Industrial Chain has not been strengthened in combination with China’s gold industry policies and security governance system. It is suggested to use the scenario analysis method to set relevant scenarios for the governance security of China’s gold Industrial Chain and make a situational judgment on its security situation. It is hoped that future research work can investigate this regard in combination with China’s gold management security policies and gold security governance system. Furthermore, combined with the machine learning algorithm and stochastic differential equation to supply and demand analysis of supply problems of China's gold Industrial Chain, targeted gold development and security governance. In addition, future research could focus on how to collect, process, and leverage IoT data more effectively. This could include developing smarter sensor technologies to provide more valuable information and advanced analytics to extract insights from large amounts of data. Moreover, this work also focuses on cross-industry cooperation and standard development. This will help establish common best practices to ensure the sustainability and security of the supply chain.

### Supplementary Information

Supplementary Information.

## Data Availability

All data generated or analysed during this study are included in this published article [and its supplementary information files]. If someone wants to request the data from this study please contact the Corresponding author.
