# Supplementary material for: Security issues of the gold industry chain based on smart blockchain in the context of the Internet of Things
Source: Sci Rep. 2024 Feb 1;14:2728. doi: 10.1038/s41598-024-52274-2 (PMC10834524; doi:10.1038/s41598-024-52274-2)
Supplement: Supplementary file 1 — Supplementary Information. [file 41598_2024_52274_MOESM1_ESM.zip › data packet/Model running environment and code running process.doc]

Hardware environment:

Computer system: 64-bit operating system, 2GB memory, 10GB disk space, USB port.

Network environment: Matching is used to synchronize and access remote servers.

Software environment:

Development language: Programming languages such as Java.

Development tool: Visual Studio Code

Dependency library and framework: Ethereum framework.

Code runtime environment: Windows 10

Running software: JetBrains PyCharm 2018.3.7 x64

Install Java and virtual environment:

Open the terminal.

Firstly, confirming whether your operating system supports the Java runtime environment is necessary. The specific steps are as follows: first, right-click on "My Computer" → select "Properties" → click on "Advanced System Settings" → select "Advanced" → click on "Environment Variables".

A new JAVA_ HOME is created in the system variable, the path is where you installed Java, and then create a new CLASSPATH variable is created with a value of: .;%JAVA_HOME%\lib;.

Finally, in the path variable, %JAVA_HOME%bin is added.

Click OK to save.

After completing the above steps, you can test whether Java has been successfully installed and configured with environment variables. java -version and javac -version are input in the terminal, if the version information can be displayed, it means that Java has successfully installed and configured environment variables.

If you need to install a virtual environment, the following command can be used: sudo apt-get install python3-venv.

Run code:

// Define a struct for the gold chain

struct GoldChain {

Blockchain blockchain;

SecureDatabase sd;

// Other struct members...

};

// Initialize the gold chain

function initGoldChain(chainId) {

// Initialize the blockchain

blockchain = initializeBlockchain(chainId);

// Initialize the secure database

sd = initializeSecureDatabase();

// Initialize the struct members of the gold chain...

return chain;

}

// Add gold asset information to the blockchain

function addGoldAsset(assetInfo) {

// Wrap the gold asset information into a block

block = createBlock(assetInfo);

// Add the block to the blockchain

blockchain.addBlock(block);

}

// Store gold asset information in the secure database

function storeGoldAssetInSD(assetInfo) {

// Encrypt the gold asset information and store it in the secure database

encryptedInfo = encryptAssetInfo(assetInfo);

sd.storeData(encryptedInfo);

}

// Retrieve gold asset information from the secure database

function getGoldAssetFromSD(assetId) {

// Retrieve the encrypted gold asset information from the secure database

encryptedInfo = sd.retrieveData(assetId);

// Decrypt the encrypted information to get the gold asset information

assetInfo = decryptAssetInfo(encryptedInfo);

return assetInfo;

}

// Check if gold asset information exists in both blockchain and secure database

function checkGoldAssetExistence(assetId) {

// Check if the block for the gold asset information exists in the blockchain

blockExists = blockchain.checkBlockExists(assetId);

// Check if the encrypted information for the gold asset exists in the secure database

dataExists = sd.checkDataExists(assetId);

// Check if the gold asset information exists in both blockchain and secure database

if (blockExists && dataExists) {

return true;

} else {

return false;

}

}

The operation process of the gold industry chain security model:

1. Initialize the gold industry chain security model: It is necessary to set the structure and parameters of the gold industry chain, including the configuration of blockchain and security databases, node communication methods, and encryption algorithm selection.
2. Create a blockchain network: Based on the set structure and parameters of the gold industry chain, a blockchain network is created, involving inter-node connections, consensus mechanism settings, etc.
3. Add gold asset information to the blockchain: The gold asset information is encapsulated into a data structure, encryption algorithms are used for encryption, and then the encrypted data is added to the blockchain.
4. Store gold asset information in a secure database: The encrypted gold asset information is stored in the secure database.
5. Check whether the gold asset information exists: By calling the functions of the blockchain and security database, it is necessary to check whether the gold asset information exists in both the blockchain and security database.
6. Implement smart contracts: JAVA and other languages are used to write smart contracts, enabling querying, trading, and pledging gold asset information.
7. Run smart contract: The smart contract is uploaded to the blockchain network and starts running.
8. Testing and debugging: The entire model is tested and debugged, encompassing the correctness of smart contracts, the security and credibility of gold asset information, etc.
9. Model deployment: After ensuring the stability and reliability of the model, the model is deployed to the actual production environment and continuously maintained and optimized.
